# Acupuncture Treatment Decreased Temporal Variability of Dynamic Functional Connectivity in Chronic Tinnitus

**DOI:** 10.3389/fnins.2021.737993

**Published:** 2022-01-28

**Authors:** Yarui Wei, Wanlin Zhang, Yu Li, Xiangwei Liu, Bixiang Zha, Sheng Hu, Yanming Wang, Xiaoxiao Wang, Xiaochun Yu, Jun Yang, Bensheng Qiu

**Affiliations:** ^1^Hefei National Lab for Physical Sciences at the Microscale and the Center for Biomedical Engineering, University of Science and Technology of China, Hefei, China; ^2^Department of Magnetic Resonance Imaging, The First Affiliated Hospital of Zhengzhou University, Zhengzhou, China; ^3^Department of Acupuncture and Rehabilitation, The First Affiliated Hospital of Anhui University of Traditional Chinese Medicine, Hefei, China; ^4^School of Medical Information Engineering, Anhui University of Traditional Chinese Medicine, Hefei, China; ^5^Institute of Acupuncture and Moxibustion, China Academy of Chinese Medical Sciences, Beijing, China

**Keywords:** acupuncture, chronic tinnitus, functional magnetic resonance imaging, dynamic functional connectivity, clustering analysis

## Abstract

Acupuncture is recommended for the relief of chronic tinnitus in traditional Chinese medicine, but the underlying neural mechanism remains unclear. The human brain is a dynamic system, and it’s unclear about acupuncture’s effects on the dynamic functional connectivity (DFC) of chronic tinnitus. Therefore, this study based on resting-state functional magnetic resonance imaging (fMRI) investigates abnormal DFC in chronic tinnitus patients and the neural activity change evoked by acupuncture treatment for tinnitus. In this study, 17 chronic tinnitus patients and 22 age- and sex-matched normal subjects were recruited, and their tinnitus-related scales and hearing levels were collected. The fMRI data were measured before and after acupuncture, and then sliding-window and k-means clustering methods were used to calculate DFC and perform clustering analysis, respectively. We found that, compared with the normal subjects, chronic tinnitus patients had higher temporal variability of DFC between the supplementary motor area and medial part of the superior frontal gyrus, and it positively correlated with hearing loss. Clustering analysis showed higher transition probability (TP) between connection states in chronic tinnitus patients, and it was positively correlated with tinnitus severity. Furthermore, the findings showed that acupuncture treatment might improve tinnitus. DFC between the posterior cingulate gyrus and angular gyrus in chronic tinnitus patients after acupuncture showed significantly decreased, and it positively correlated with the improvement of tinnitus. Clustering analysis showed that acupuncture treatment might promote chronic tinnitus patients under lower DFC state, and it also positively correlated with the improvement of tinnitus. This study suggests that acupuncture as an alternative therapy method might decrease the tinnitus severity by decreasing the time variability of DFC in chronic tinnitus patients.

## Introduction

Tinnitus is defined as the perception of non-speech sound in the absence of an external acoustic stimulus. It is a common medical symptom that can be debilitating, and approximately 1 in 10 adults have had the experience of tinnitus (Bhatt et al., 2016; Yang et al., 2018). However, no effective drug therapy is available for the tinnitus (Baguley et al., 2013), and various treatment options are applied, including education and counseling, sound therapy, cognitive behavioral therapy (CBT), medications, dietary supplementation, and acupuncture (Tunkel et al., 2014; Lv et al., 2021).

Acupuncture is recommended for the relief of chronic tinnitus in traditional Chinese medicine. Although some studies reported that acupuncture had few effective treatments for tinnitus (Podoshin et al., 1991; Kim et al., 2012), recent studies showed an effective acupuncture treatment for tinnitus (Pang et al., 2019; Huang et al., 2021; Wang et al., 2021). The acupoints commonly used are *Tinggong* (SI 19), *Tinghui* (GB 2), *Yifeng* (TE 17), *Shuaigu* (GB 8), *Ermigen* (R 2), *Fengchi* (GB 20), *Zhongzhu* (TE 3), *Luxi* (TE 19), and *Ermen* (TE 21). Moreover, the researchers assessed the effect of *deqi* for patients who received acupuncture to alleviate tinnitus by the randomization procedure, large sample, and standardized protocol (Xie et al., 2014). Lin et al. (2019) explored factors influencing the efficiency of acupuncture in tinnitus patients and found that the combination of acupoints and the number of acupuncture sessions contributed to a considerably better outcome.

Some studies were aimed to explore the science evidence of acupuncture in the treatment of tinnitus patients. Due to the lack of objective marker of tinnitus, tinnitus-related scales were used to assess the effect of acupuncture (Jeon et al., 2012; Kim et al., 2017). The acupuncture’s effects for tinnitus were associated with the improvement of cochlear blood flow via the infrared thermography test (Cai et al., 2019). A previous study also assessed the cochlear function in patients with tinnitus by analyzing otoacoustic emissions and found that the amplitude of otoacoustic emissions was increased after acupuncture (de Azevedo et al., 2007). Ethyl single-photon emission computed tomography (^99m^Tc-ECD SPECT) was also used to investigate the effect of acupuncture on brain perfusion in tinnitus patients; however, no between-group brain perfusion differences were observed (Laureano et al., 2016). Therefore, in terms of neural correlate, the science evidence of acupuncture for treating tinnitus is lacking. The resolution of functional magnetic resonance imaging (fMRI) is better than that of SPECT, and fMRI is a non-invasive functional imaging method. The previous study explored the central mechanism of transauricular vagus nerve stimulation (taVNS) to normal human by fMRI and found a suitable taVNS site for potential tinnitus treatment (Peng et al., 2018).

It is noteworthy that both the appearance of tinnitus and the effect of acupuncture tightly associate with the central system. The researchers explored the functional anatomy of tinnitus and found plastic changes in multiple neural systems in tinnitus patients (Lockwood et al., 1998). The previous studies showed the abnormal brain activity among the auditory cortex and non-auditory regions (limbic system, subcortex regions, brainstem, and cerebellum) in tinnitus patients (Liu and Chen, 2012; Bauer et al., 2013; Chen et al., 2015a,^2017^; Berlot et al., 2020; Marschall et al., 2021). Moreover, researchers found that acupuncture modulated the cerebral cortex, limbic system, brainstem, cerebellum, and subcortical gray structures of human brain and evoked the integrated response of these regions (Hui et al., 2000; Hui et al., 2005; Napadow et al., 2009). Manual acupuncture at acupoint *Hegu* (LI 4) produced prominent decreases of fMRI signals in the limbic system and subcortical structures and increases of fMRI signals in the somatosensory cortex (Hui et al., 2000).

Static functional connectivity (SFC) analysis based on resting state fMRI investigated abnormal functional connectivity (FC) among different brain areas in tinnitus patients (Lv et al., 2017; Feng et al., 2018; Lan et al., 2020). Researchers have found some intrinsic connectivity networks using the SFC analysis, such as default mode network (Greicius et al., 2009), ventral and dorsal attention networks (Fox et al., 2006), and salience network (Seeley et al., 2007). However, the SFC is based on the implicit assumption of spatial and temporal stationary of fMRI data, which is over simple for complex activities of the human brain (Allen et al., 2014). When the mental activity is unconstrained, dynamics are potentially even more prominent under the resting state. Evidences suggested that dynamic functional connectivity (DFC) supply us new information about abnormal FC on the brain of patients with various diseases (Demirtas et al., 2016; Guo et al., 2018; Li et al., 2019; Wang et al., 2020; Chen et al., 2020a,b). However, the abnormal DFC and the neural activity change after acupuncture treatment were unclear in tinnitus patients.

In the present study, we aimed to investigate acupuncture therapy’s effects on the DFC of the tinnitus patients. We analyzed the temporal variability of DFC on chronic patients before and after acupuncture and classified and assessed the states of DFC using k-means clustering analysis (Allen et al., 2014; Wang et al., 2016; Li et al., 2019). To our best knowledge, this study is the first to explore abnormal DFC in chronic tinnitus patients and the neural mechanism of acupuncture treatment for tinnitus.

## Materials and Methods

### Participants

To begin, we collected 33 tinnitus patients and 22 normal subjects from December 2017 to October 2019. In these patients, we excluded six acute tinnitus patients, and 10 tinnitus patients withdrew consent. Finally, 17 chronic tinnitus patients and 22 aged- and sexed-matched normal subjects were included in this study (Table 1). The inclusion criteria of chronic tinnitus patients were as follows: (i) between 18 and 70 years of age, right-handed; (ii) subjective neurological tinnitus, patients reported that they heard sound on the unilateral or bilateral tinnitus, no history of acupuncture treatment; (iii) no magnetic resonance contraindications; (iv) no medical history with congenital or hereditary disease, endocrine immune disease, mental or neurological system disease or the severe disease of heart, liver, or kidney, and so on. The exclusion criteria of chronic tinnitus patients were as follows: (i) left-handed; (ii) other subjective tinnitus, objective tinnitus (including mechanical or pulsatile tinnitus), or acute tinnitus; (iii) magnetic resonance contraindications; (iv) pregnant, breastfeeding, or menstruating; (v) medical history with congenital or hereditary disease, endocrine immune disease, mental or neurological system disease, or the severe disease of heart, liver, or kidney, and so on; (vi) the history of mental or neurological medicine. In addition, all patients were instructed to avoid starting other interventions. All subjects gave the informed consent, and this study was approved by the Ethics Committee of the First Affiliated Hospital of Anhui University of Traditional Chinese Medicine (ethics number: 2021AH-29).

**TABLE 1:** The demographic data of chronic tinnitus patients and normal subjects.

	Chronic tinnitus (*n* = 17)	Control group (*n* = 22)	χ^2^*/t*-value	*P*-value
Age (years)	47.29 ± 2.85	46.82 ± 2.37	–0.129	0.898
Sex (M/F)	7/10	6/16	0.834	0.283
Handedness (R/L)	17/0	22/0	–	–
Tinnitus duration (years)	8.38 ± 2.99	–	–	–
Tinnitus location (L/R/B)	4/3/10	–	–	–

*B, bilateral; F, female; L, left; M, male; R, right.*

### Tinnitus-Related Scales and Hearing Level

The scales to assess tinnitus severity included tinnitus handicap inventory (THI) (Newman et al., 1996), tinnitus disturbance inventory (TDI) (Baguley et al., 2000), visual analog scales (VAS) (Lindberg et al., 1992), and Khalfa hyperacusis questionnaire (KHQ) (Khalfa et al., 2002). TDI also described the tinnitus loudness. TDI divides the degree of the tinnitus loudness into seven levels, namely zero to sixth levels. Zero level means no tinnitus, and the sixth level means that the tinnitus is extremely loud and unbearable. Pure tone audiometry was used to measure the hearing threshold for chronic tinnitus patients before and after acupuncture. Before acupuncture, tinnitus-related scales were collected for all chronic tinnitus patients with the data of hearing level for 15 of the patients; after acupuncture, tinnitus-related scales were collected for 14 chronic tinnitus patients with the data of hearing level for 11 of the patients.

### Acupuncture Treatment

Acupuncture treatment was performed by specialized and licensed acupuncturists (Jun Yang and Bixiang Zha). The disposable sterile needle of 0.35 mm in diameter and 40 mm in length (Suzhou Tianxie Acupuncture and Moxibustion Appliance Co., Ltd., China) was used for performing manual acupuncture. The 7 obligatory acupoints were SI 19, GB 2, TE 17, GB 8, R 2, GB 20, TE 3 on the ipsilateral side with tinnitus. Additional acupoints were selected according to meridian diagnosis and the patient’s symptoms. After sterilization, the acupuncturist manipulated the needle within the tolerance of the subject until the subjects obtained the *deqi* sensation without sharp pain. After achieving *deqi* sensation, the needle was located on the acupoints for 30 min. Chronic tinnitus patients received 12 sessions of acupuncture treatments over 4 weeks (3 times per week). Fourteen chronic tinnitus patients finished the 12 sessions of acupuncture treatment among 17 chronic tinnitus patients. The patients didn’t receive other supplementary medicine, electroacupuncture, or moxibustion throughout the course of acupuncture.

### Data Acquirement

All subjects were scanned using a 3.0 T MRI scanner (Discovery MR750, GE, United States) with an 8-channel receiver array head coil located in Information Science Laboratory Center of University of Science and Technology of China. Head motion and scanner noise were reduced using foam padding and earplugs. The subjects were instructed to lie on their back quietly with their eyes closed but not to fall asleep and avoid thinking of anything particular during the scanning. We collected the data of MRI for all chronic tinnitus patients before acupuncture and for 12 of the patients after acupuncture. Among the 12 patients, we collected the data of tinnitus-related scales for all chronic tinnitus patients and the data of hearing level for 11 of the patients before acupuncture, and the data of tinnitus-related scales for 11 of the patients and the data of hearing level for 10 of the patients after acupuncture. The data of MRI after acupuncture was conducted 2 days after a course of acupuncture treatment. Structure images were acquired with 3D T1 BRAVO sequence with the following settings: repetition time (TR)/echo time (TE) = 8,160/31.8 ms, slice number = 188, slice thickness = 1 mm, slice gap = 0 mm, flip angle = 12°, field of view (FOV) = 25.6 × 25.6 cm^2^, number of averages = 1, matrix size = 256 × 256, voxel size = 1 × 1 × 1 mm^3^. Functional images were acquired transversely with gradient spin echo planar imaging sequence with the following settings: TR/TE = 2,400/30 ms, slice number = 46, slice thickness = 3 mm, slice gap = 3 mm, flip angle = 90°, FOV = 19.2 × 19.2 cm^2^, number of averages = 1, matrix size = 64 × 64, voxel size = 3 × 3 × 3 mm^3^. A total of 217 volumes were collected, resulting in a total scan time of 520.8 s.

### Data Preprocessing

Data analyses were preprocessed using Data Processing Assistant for Resting-State fMRI (DPARSF) programs (Yan and Zang, 2010), which is based on Statistical Parametric Mapping (SPM8)^1^ and MATLAB (MathWorks). The first 5 volumes were discarded and the remaining 212 consecutive volumes were used for data analysis. Slice-timing and realignment for head motion correction were performed. Nuisance covariates were regressed [including Friston 24 head motion parameters (Friston et al., 1996) and white matter and cerebrospinal fluid signals]. Then, data were coregistered with the structural images, spatial normalized to the Montreal Neurological Institute template (resampling voxel size = 3 × 3 × 3 mm^3^), smoothed with an isotropic Gaussian kernel [full width at half maximum (FWHM) = 6 mm], and detrended and filtered (0.01∼0.08 Hz). Image volumes with framewise displacement (FD) > 0.2 mm were scrubbed to reduce the effect of head motion using spline interpolation (He et al., 2018).

### Regions of Interest Selection and Static Functional Connectivity Network Analysis

We used the automated anatomical labeling (AAL) template to calculate FC based on region of interest (ROI) analysis. A total of 90 ROIs of AAL template (without cerebellum) were divided into six main regions (including prefrontal regions, other regions of frontal lobe, parietal regions, occipital regions, temporal regions, and subcortical regions) (Tzourio-Mazoyer et al., 2002).

The average time courses were extracted from 90 ROIs for calculating SFC. Later, we calculated Pearson’s correlation coefficients between each pair of the averaged time course in 90 ROIs, then Fisher *z*-transformation was used to convert *r* values into *z* values to improve the normality of correlation distribution.

### Dynamic Functional Connectivity Network Analysis

Sliding window approach was widely used to measure brain DFC network for each subject (Allen et al., 2014; Zalesky et al., 2014). Here, we constructed a sliding temporal window (Hamming window) of 12 TRs. Following that, this window was used to slide on the time course with a step of 1 TR (2.4 s). In total, 201 (212–12+1) temporal windows were produced. We then calculated FC in accordance with SFC analysis in each window, resulting in a time series of FC matrices (90 × 90, Fisher *z*-transformed) for the next analysis.

Unlike SFC, DFC reflected time-varying FC network. Therefore, we employed three common metrics to describe its dynamic characteristics: DFC-Str (strength) (Liu et al., 2018), DFC-SD (standard deviation) (Li et al., 2019), and ALFF-FC (amplitude of low frequency fluctuation of FC) (Han et al., 2011). Different metrics might reflect different temporal variability of DFC and complement each other. DFC-Str is the average of DFC, and DFC-SD is the standard deviation for the time series of FC matrices. ALFF-FC is a measure assessing the fluctuation of FC within the low-frequency range. A high-pass filtering for the original BOLD signals and a low-pass filtering for correlation coefficient time series with the cut-off frequency 1/*w* are suggested to remove spurious fluctuations in DFC, when a certain window size *w* is given (Leonardi and Van De Ville, 2015). Hence, for a given window size *w* (12 × 2.4 s = 28.8 s), we high-pass filtered the ROI signals with cut-off frequency 1/*w* prior to calculation of connectivity matrices, and then low-pass filtered the correlation coefficient time series with cut-off frequency 1/*w* (Qin et al., 2015). Accordingly, ALFF-FC values were calculated within the frequency band of DFC from 0 to 1/*w*.

### Clustering Analysis

To estimate the frequency and structure of DFC patterns, we applied a k-means algorithm (Lloyd, 1982) with the L1 distance function (Manhattan distance) (Allen et al., 2014) to cluster DFC data. For each subject, 201 windows × 4,005 features were offered to the clustering analysis. We performed twice clustering analysis, one for chronic tinnitus patients before acupuncture and normal subjects and one for chronic tinnitus patients before and after acupuncture. Similar to EEG microstate analysis (Pascual-Marqui et al., 1995), for reducing redundancy between time windows (the chosen time step of 1 TR induces high autocorrelation in FC time series) and decreasing computational load, we performed subsampling across the temporal dimension to identify windowed covariance matrices with local maxima in FC variance (Allen et al., 2014; Wang et al., 2016). This yielded 1,435 exemplars [mean: 1,435/(17+22) = 36.8 windows per subjects; range: 29∼44] for the first clustering analysis and 867 exemplars [mean: 867/(12+12) = 36.1 windows per subjects; range: 29∼42] for the second clustering analysis, then k-means were adopted for these exemplars with 500 repetitions to escape local minima, and the centroids produced from clustering exemplars were used to initialize clustering of all data (chronic tinnitus patients before acupuncture and normal subjects: [(17+22) subjects × 201 windows = 7,839 instances; chronic tinnitus patients before and after acupuncture: (12+12) subjects × 201 windows = 4,824 instances]. Finally, the optimal number of clusters ranging from 2 to 20 was estimated according to the elbow criterion (Liu et al., 2017), and the cluster medians (reshaped to matrix) were regarded as the FC state.

### State Analysis

To compare state configuration between groups and within group, we computed one metric between states: transition probability (TP) (Li et al., 2019). TP was computed as the probability of transitions from one state to other state or lasting in the same state. When N cluster centers are calculated in the previous step, there will be N × N kinds of transition between states.

### Statistical Analysis

To identify whether the demographic and scale data of each group were normally distributed, we performed Shapiro-Wilk’s test. Results showed TDI scores were not normally distributed in chronic tinnitus before acupuncture, other demographic and scale data of each group were normally distributed. Two-sample *t*-tests and χ^2^-tests were used to compare demographic data between chronic tinnitus patients and normal subjects, and paired Student’s *t*-tests was used to compare the effective of acupuncture treatment within chronic tinnitus patients before and after acupuncture with SPSS software. Wilcoxon-Signed-Rank test was used to compare TDI scores within chronic tinnitus patients before and after acupuncture. For SFC values and all metrics extracted from DFC (including DFC-Str, DFC-SD, ALFF-FC, and TP), we performed a general linear model (GLM) to evaluate between-group and within-group differences in age, sex, and mean FD as covariates in the GRETNA toolbox (Wang et al., 2015). Afterward, multiple comparison correction (false discovery rate, FDR) was performed for connection metrics (SFC, DFC-Str, DFC-SD, and ALFF-FC). We calculated Pearson’s correlation coefficients to perform correlation analysis between tinnitus-related scales scores (excluding TDI that used Spearmen’s correlation) or hearing level and significant results between groups or within group.

## Results

### Tinnitus Severity and Hearing Level Before and After Acupuncture

The scores of TDI and VAS were significantly decreased in chronic tinnitus patients after acupuncture compared with before acupuncture (TDI: *Z* = –2.251, *P* < 0.05; VAS: *T* = –3.040, *P* < 0.01, Table 2). Any other significant difference was found in neither other tinnitus-related scales nor hearing level in chronic tinnitus patients after acupuncture compared with before acupuncture (Table 2).

**TABLE 2:** Tinnitus severity and hearing level before and after acupuncture.

	Pre-acupuncture	Post-acupuncture	*T/Z-*value	*P*-value
Left ear (dB/SPL, *n* = 11)	30.45 + 3.96	29.89 + 3.30	–0.429	0.677
Right ear (dB/SPL, *n* = 11)	38.21 + 7.63	39.25 + 6.78	0.599	0.562
THI (*n* = 14)	52.00 + 7.14	46.71 + 7.25	–1.352	0.199
TDI (*n* = 14)	3.07 + 0.51	2.43 + 0.36	–2.251	0.024*
VAS (*n* = 14)	5.64 + 0.59	4.50 + 0.50	–3.040	0.009*
KHQ (*n* = 14)	48.43 + 6.17	43.36 + 5.95	–1.533	0.149

dB, deci Bel; KHQ, Khalfa hyperacusis questionnaire; SPL, sound pressure level; TDI, tinnitus disturbance inventory; THI, tinnitus handicap inventory; VAS, visual analog scales. *P < 0.05.

### Static Functional Connectivity

**FIGURE 1:**
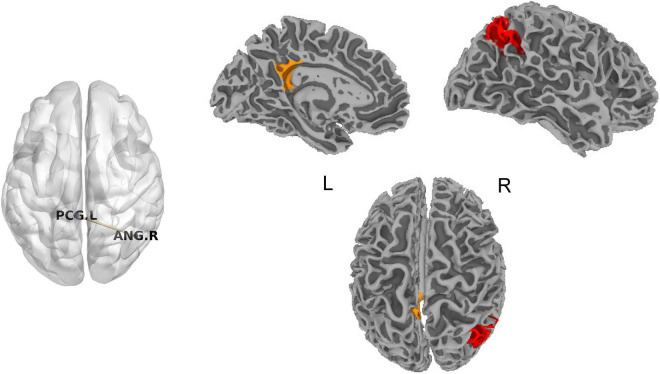
Significant difference for SFC in chronic tinnitus patients before and after acupuncture. ANG, angular gyrus; L, left; PCG, posterior cingulate gyrus; R, right; SFC, static functional connectivity.

No significant difference for SFC was found in chronic tinnitus patients before acupuncture compared with normal subjects after FDR correction. SFC between the left posterior cingulate cortex (PCC) and right angular gyrus (ANG) in chronic tinnitus patients after acupuncture was decreased compared with before acupuncture (*T* = -8.03, *P* = 0.0000114, Figure 1). No significant correlation was found between SFC of the left PCC and right ANG and tinnitus-related scales scores or hearing level.

### Dynamic Functional Connectivity

#### Chronic Tinnitus Patients Before Acupuncture and Normal Subjects

**FIGURE 2:**
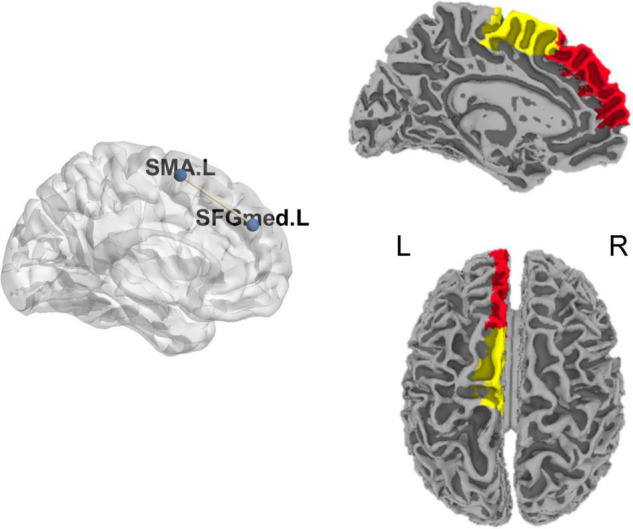
Significant difference for DFC between chronic tinnitus patients before acupuncture and normal subjects. DFC, dynamic functional connectivity; L, left; R, right; SFGmed, superior frontal gyrus, medial part; SMA, supplementary motor area.

Based on two metrics (DFC-SD and ALFF-FC) to describe the time variability of DFC, higher DFC between the left supplementary motor area (SMA) and medial part of the left superior frontal gyrus (SFGmed) was found in chronic tinnitus patients before acupuncture than that in normal subjects (Table 3 and Figure 2). Based on the metrics (DFC-Str), no significant different DFC was found in chronic tinnitus patients before acupuncture than that in normal subjects.

**TABLE 3:** Comparisons of temporal variability between chronic tinnitus patients before acupuncture and normal subjects.

DFC parameter	DFC pair	*T*-value	*P-*value
DFC-SD	SMA.L-SFCmed.L	5.42	0.0000074
ALFF-FC	SMA.L-SFCmed.L	5.24	0.0000106

*ALFF, amplitude of low frequency fluctuation; DFC, dynamic functional connectivity; FC, functional connectivity; L, left; SD, standard deviation; SFGmed, superior frontal gyrus, medial part; SMA, supplementary motor area.*

**FIGURE 3:**
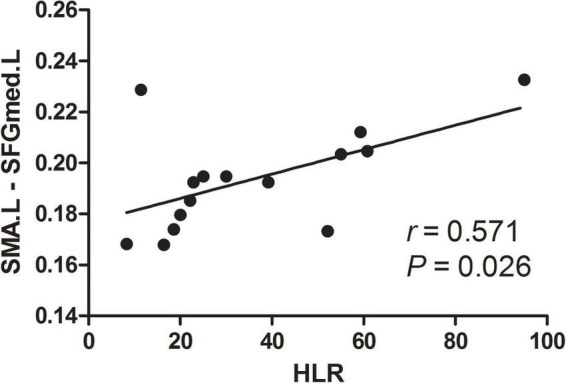
Correlation between abnormal DFC and tinnitus severity. HLR, hearing level of the right ear; L, left; SFGmed, superior frontal gyrus, medial part; SMA, supplementary motor area.

To further explore the relationship between tinnitus severity and temporal variability of DFC in chronic tinnitus patients, we correlated tinnitus-related scales scores and hearing level with significant results from DFC-SD and ALFF-FC using the Pearson’s or Spearman’s (TDI scores) correlation coefficient. ALFF-FC between the left SMA and left SFGmed positively correlated with the hearing level of the right ear in chronic tinnitus patients before acupuncture (*r* = 0.571, *P* = 0.026, uncorrected, Figure 3). No other significant correlation was found between DFC of the left SMA and left SFGmed and other tinnitus-related scales or hearing level.

#### Chronic Tinnitus Patients Before and After Acupuncture

**FIGURE 4:**
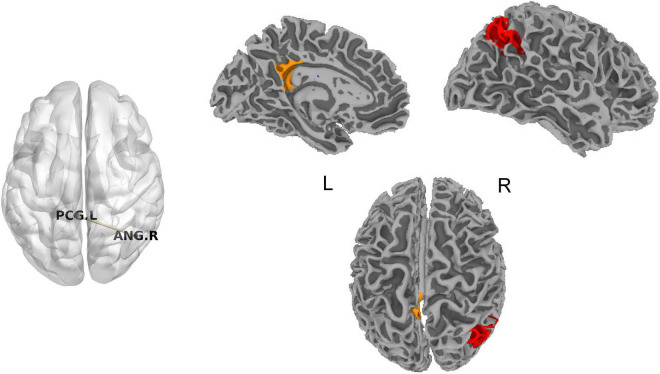
Significant difference for DFC in chronic tinnitus patients before and after acupuncture. ANG, angular gyrus; DFC, dynamic functional connectivity; L, left; PCG, posterior cingulate gyrus; R, right.

Based on the metric (DFC-Str) to describe DFC, DFC between the left PCC and right ANG was decreased in chronic tinnitus patients after acupuncture compared with before acupuncture (*T* = -8.96, *P* = 0.0000043, Figure 4). Based on the metrics (DFC-SD and ALFF-FC) to describe the time variability of DFC, no significant difference for DFC was found in chronic tinnitus patients after acupuncture compared with before acupuncture.

To further explore the relationship between tinnitus severity and temporal variability of DFC in chronic tinnitus patients before and after acupuncture, we correlated tinnitus-related scales scores and hearing level with significant results from DFC-Str using the Pearson’s or Spearman’s (TDI scores) correlation coefficient. The decreased DFC-Str between the left PCC and left ANG positively correlated with the decreased TDI scores in chronic tinnitus patients before and after acupuncture (*r*_*s*_ = 0.623, *P* = 0.044, uncorrected). No other significant correlation was found between DFC-Str of the left PCC and left ANG and other tinnitus-related scales scores or hearing level.

### Clustering and State Analysis

#### Chronic Tinnitus Patients Before Acupuncture and Normal Subjects

**FIGURE 5:**
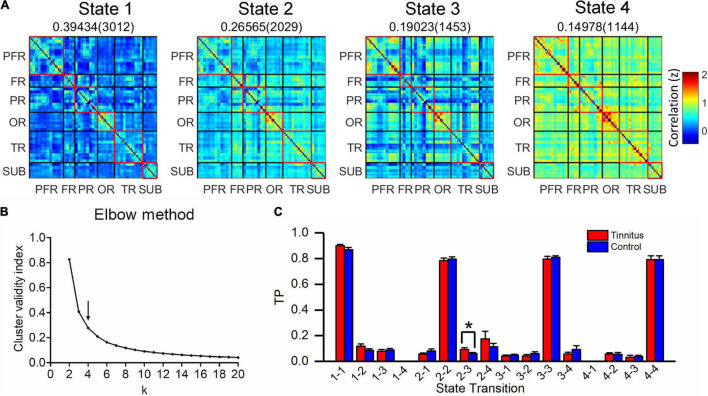
Clustering analysis of four states in chronic tinnitus patients before acupuncture and normal subjects. **(A)** Four states (centroids) from clustering analysis. The total number (in brackets) and proportion of occurrences are listed above each state. **(B)** Elbow criterion. The arrow represents the optimal *k* value. *k*, the number of clusters; Cluster validity index, calculating as the ratio of within-cluster distance to between-cluster distance. **(C)** Comparison of TP between chronic tinnitus patients before acupuncture and normal subjects. FR, other regions of frontal lobe; PR, parietal regions; OR, occipital regions; PFR, prefrontal regions; SUB, subcortex regions; TP, transition probability; TR, temporal regions. *P < 0.05.

Based on elbow criterion, four clusters (k) were determined in a search window ranging from 2 to 20 (Figures 5A,B). For each pair condition, we compared TP between groups. Compared with normal subjects, we found that the TP from state 2 to state 3 in chronic tinnitus patients before acupuncture was higher (*T* = 2.303, *P* = 0.028, uncorrected, Figure 5C).

**FIGURE 6:**
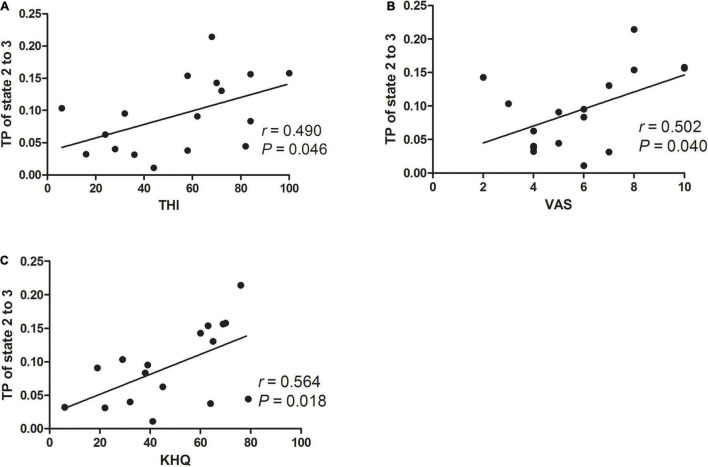
Correlation between TP from state 2 to state 3 and tinnitus severity in chronic tinnitus patients before acupuncture. We found that the TP from state 2 to state 3 in chronic tinnitus patients before acupuncture positively correlated with THI **(A)**, VAS **(B)**, and KHQ **(C)**. KHQ, Khalfa hyperacusis questionnaire; THI, tinnitus handicap inventory; TP, transition probability; VAS, visual analog scales.

Meanwhile, for exploring the association between state configuration and tinnitus severity in chronic tinnitus patients before acupuncture, we performed Pearson or Spearman’s (TDI scores) correlation analysis between TP from state 2 to state 3 and tinnitus-related scales scores or hearing level. As shown in Figure 6, TP from state 2 to state 3 in chronic tinnitus patients before acupuncture positively correlated with tinnitus severity (THI: *r* = 0.490, *P* = 0.046, uncorrected; VAS: *r* = 0.502, *P* = 0.040, uncorrected; KHQ: *r* = 0.564, *P* = 0.018, uncorrected). No other significant correlation was found between TP from state 2 to state 3 and other tinnitus-related scales scores or hearing level.

#### Chronic Tinnitus Patients Before and After Acupuncture

**FIGURE 7:**
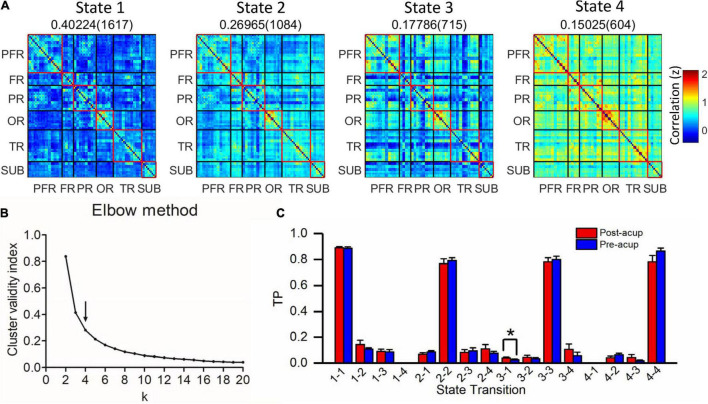
Clustering analysis of four states in chronic tinnitus patients before and after acupuncture. **(A)** Four states (centroids) from clustering analysis. The total number (in brackets) and proportion of occurrences are listed above each state. **(B)** Elbow criterion. The arrow represents the optimal *k* value. *k*, the number of clusters; Cluster validity index, calculating as the ratio of within-cluster distance to between-cluster distance. **(C)** Comparison of TP within chronic tinnitus patients before and after acupuncture. FR, other regions of frontal lobe; PR, parietal regions; OR, occipital regions; PFR, prefrontal regions; SUB, subcortex regions; TP, transition probability; TR, temporal regions. *P < 0.05.

Based on elbow criterion, four clusters (k) were determined in a search window ranging from 2 to 20 (Figures 7A,B). For each pair condition, we compared TP within chronic tinnitus patients before and after acupuncture. Compared with before acupuncture, we found that the TP from state 3 (hyper-connected pattern) to state 1 (hypo-connected pattern) in chronic tinnitus patients after acupuncture was increased (*T* = 2.586, *P* = 0.027, uncorrected, Figure 7C).

For exploring the association between state configuration and tinnitus severity in chronic tinnitus patients before and after acupuncture, we performed Pearson’s or Spearman’s (TDI scores) correlation analysis between TP from state 3 to state 1 and tinnitus-related scales scores. Combining the data in chronic tinnitus patients before and after acupuncture, TP from state 3 to state 1 negatively correlated with TDI scores (*r*_*s*_ = -0.416, *P* = 0.048, uncorrected). No other significant correlation was found between TP from state 3 to state 1 and other tinnitus-related scales scores or hearing level.

## Discussion

This study explores the brain functional network of chronic tinnitus patients and the neural mechanism of acupuncture treatment for chronic tinnitus. The findings suggest that acupuncture might improve tinnitus by decreasing the temporal variability of DFC of the patients. The result showed that, compared with normal subjects, chronic tinnitus patients suffered a higher temporal variability of DFC between the left SMA and left SFGmed, which positively correlated with hearing loss of the right ear. Moreover, clustering analysis showed higher TP between brain connection states in chronic tinnitus patients, which was positively correlated with tinnitus severity. Consistent with recent studies (Cai et al., 2019; Pang et al., 2019; Kuzucu and Karaca, 2020), the acupuncture treatment effectively improved tinnitus. DFC between the left PCG and right AG in chronic tinnitus patients after acupuncture were observed to significantly decrease, and the amount was positively correlated with the improvement of tinnitus. Clustering analysis showed acupuncture treatment might promote chronic tinnitus patients under lower DFC state, which was also positively correlated with the improvement of tinnitus.

This study found higher temporal variability of DFC between the SMA and SFGmed in chronic tinnitus patients. The previous studies indicated that the SMA and the SFGmed tightly associated with tinnitus (Chen et al., 2016, Cheng et al., 2020). The previous study showed that effective connectivity from the SFG to the SMA was higher in chronic tinnitus patients compared with normal subjects (Chen et al., 2016). Moreover, the meta-analysis showed that tinnitus patients showed structural abnormality in the SMA and the SFG (Cheng et al., 2020). The abnormal activity in the SFG for tinnitus patients might correlate with the emotional problem produced by tinnitus (Golm et al., 2013). Moreover, the previous study suggested that repetitive transcranial magnetic stimulation (rTMS) of the auditory cortex for normal subjects produced the brain activation of the motor cortex (Lee et al., 2013), and rTMS on motor cortex plasticity for a large sample of tinnitus patients decreased short-interval intra-cortical inhibition that might reflect modulation of GABAergic mechanisms directly or indirectly related to rTMS treatment effects (Schecklmann et al., 2014). Therefore, the change of brain functional network between the SMA and SFGmed might be useful to guide the rTMS treatment for tinnitus. Higher time variability of DFC between the left SMA and left SFGmed and its association with hearing loss in chronic tinnitus patients suggest that decreased time variability of DFC might improve the hearing level of chronic tinnitus patients.

The result in this study showed that the temporal variability of DFC between the left PCG and right AG was significantly decreased in chronic tinnitus patients after acupuncture, which was positively correlated with the improvement of tinnitus. Acupuncture might decrease the time variability of DFC between the left PCG and right AG in chronic tinnitus patients that positively correlated with the improvement of tinnitus (TDI scores). This study found TDI and VAS scales scores decreased in chronic tinnitus patients after acupuncture, which is consistent with the previous study (Kim et al., 2017; Kuzucu and Karaca, 2020). The above results indicated that the neural mechanism of acupuncture treatment improving tinnitus might be decreasing the time variability of DFC between the left PCG and right AG. The previous studies also indicated that the PCG and the AG tightly associated with chronic tinnitus (Chen et al., 2014, 2015b; Krick et al., 2017). Impaired visual attention function was found in chronic tinnitus patients, and Heidelberg Neuro-Music Therapy treatment helped shift the attention from the auditory phantom percept toward visual cues in chronic tinnitus patients through the AG (Krick et al., 2017). Moreover, the spontaneous brain activity of the right AG, SFG, and medial temporal gyrus in chronic tinnitus patients was higher than that in normal subjects and correlated with tinnitus severity (Chen et al., 2014). Tinnitus patients showed stronger activations to tinnitus-related sentences in comparison to neutral sentences than healthy controls in various limbic/emotional processing areas, such as the anterior cingulate cortex, middle cingulate cortex, PCG, retrosplenial cortex and insula and also in frontal areas, and the fronto-parietal-cingulate network associated with tinnitus-related distress (Golm et al., 2013). Meanwhile, researchers indicated that tinnitus duration and distress were associated with brain areas (right inferior frontal gyrus, right ventro-medial prefrontal lobe, and right PCG) involved in attentional and emotional processing (Schecklmann et al., 2013). The results in this study suggest that acupuncture might modulate attentional- and emotional-related brain areas to decrease the tinnitus severity.

It’s noteworthy that the clustering analysis of DFC suggests that acupuncture treatment may improve tinnitus under hypo-connected pattern. Higher TP from state 2 to state 3 was found in chronic tinnitus patients before acupuncture compared with normal subjects, and increased TP from state 3 (hyper-connected pattern) to state 1 (hypo-connected pattern) was found in chronic tinnitus patients after acupuncture compared with before acupuncture. No significant difference of TP from state 2 to state 3 was found in chronic tinnitus patients after acupuncture compared with before acupuncture. However, acupuncture might improve tinnitus by transitioning state 3 (hyper-connected pattern) to state 1 (hypo-connected pattern). Combining the data of chronic tinnitus patients before and after acupuncture, we found TP from state 3 to state 1 negatively correlated with the tinnitus severity.

This study helps find the objective indicator to assess tinnitus by comparing the brain activity between chronic tinnitus and normal subjects and within chronic tinnitus patients before and after acupuncture. Compared with SFC, DFC may be more suitable as an objective indicator for assessing tinnitus. Compared with normal subjects, the time variability of DFC between the left SMA and left SFGmed in chronic tinnitus patients was higher, which positively correlated with hearing loss of the right ear. Moreover, DFC between the left PCG and right AG in chronic tinnitus patients after acupuncture showed as significantly decreased, which was positively correlated with the improvement of tinnitus. No significant difference for SFC after FDR correction was found between chronic tinnitus patients before acupuncture and normal subjects, which is inconsistent with previous studies (Chen et al., 2015a; Hinkley et al., 2015; Lv et al., 2017). This inconsistency might be attributable to a variety of factors: ROIs selection, heterogeneous samples, and different preprocessing methods. Instead of using the AAL atlas, seeds in many studies were defined by prior knowledge (Chen et al., 2015a), some tinnitus-related areas using other atlas (Hinkley et al., 2015; Chen et al., 2017), or independent component analysis (Leaver et al., 2016). Although SFC between the left PCC and left ANG in chronic tinnitus patients after acupuncture was also decreased compared with before acupuncture, no significant correlation was found between SFC of the left PCC and left ANG and tinnitus-related scales scores or hearing level.

There are some limitations in this study. First, the sample size is small. In fact, we spent 2 years (from December 2017 to October 2019) to recruit 33 tinnitus patients and 22 normal subjects. In these patients, we excluded six acute tinnitus patients, and 10 tinnitus patients withdrew consent. Therefore, chronic tinnitus patients in this study were well-characterized. Second, no sham treatment or other interventions for normal subjects and chronic tinnitus patients was designed. The result in this study showed that the temporal variability of DFC between the left PCG (belonging to the limbic system) and right AG was significantly decreased in chronic tinnitus patients after acupuncture, which was positively correlated with the improvement of tinnitus. Moreover, the previous study showed that acupuncture modulated the limbic system in healthy subjects (Hui et al., 2000), which is consistent with the findings in our study. On the other hand, there are significant problems with sham interventions and how they are applied in trials of acupuncture, which is caused by paucity of physiological investigations of acupuncture (Birch et al., 2022). Acupuncture is a clinical subject that is safe and effective for the treatment of disease, which has been confirmed by many large authoritative clinical observations (Chang et al., 2019; He et al., 2020). In this study, we compared the FC of chronic tinnitus with normal subjects and showed preliminary results for acupuncture treatment of chronic tinnitus. Future studies should increase the sample size and design sham acupuncture group to make the effectiveness of acupuncture treatment for tinnitus more reliable.

## Conclusion

As an alternative therapy method, acupuncture might improve tinnitus by decreasing the time variability of DFC in chronic tinnitus patients. Furthermore, this study might provide the neurophysiological evidence for acupuncture treatment of tinnitus clinically.

## Data Availability Statement

The original contributions presented in the study are included in the article, further inquiries can be directed to the corresponding author/s.

## Ethics Statement

The studies involving human participants were reviewed and approved by the Ethics Committee of the First Affiliated Hospital of Anhui University of Traditional Chinese Medicine. The patients/participants provided their written informed consent to participate in this study.

## Author Contributions

BQ, JY, XY, and YRW contributed to the conception of the study. YRW, WZ, XL, and YMW performed the experiment. YRW and YL performed the data analyses. YRW wrote the manuscript. BZ, SH, XW, XY, JY, and BQ helped perform the analysis with constructive discussions. All authors contributed to the article and approved the submitted version.

## Conflict of Interest

The authors declare that the research was conducted in the absence of any commercial or financial relationships that could be construed as a potential conflict of interest.

## Publisher’s Note

All claims expressed in this article are solely those of the authors and do not necessarily represent those of their affiliated organizations, or those of the publisher, the editors and the reviewers. Any product that may be evaluated in this article, or claim that may be made by its manufacturer, is not guaranteed or endorsed by the publisher.

## Footnotes

1
http://www.fil.ion.ucl.ac.uk/spm
